# Measurement of crosstalk in stereoscopic display systems used for vision research

**DOI:** 10.1167/16.15.14

**Published:** 2016-12-15

**Authors:** Daniel H. Baker, Milena Kaestner, André D. Gouws

**Affiliations:** daniel.baker@york.ac.uk http://www.york.ac.uk/psychology/staff/academicstaff/daniel/; mk643@york.ac.uk http://www.york.ac.uk/psychology/staff/postgrads/mk643/; andre.gouws@ynic.york.ac.uk https://www.ynic.york.ac.uk/about-us/people/andre; Department of Psychology, University of York, York, UK; Department of Psychology, University of York, York, UK; Department of Psychology, University of York, York, UK

**Keywords:** *crosstalk*, *stereo vision*, *ghosting*, *anaglyph*, *3-D displays*

## Abstract

Studying binocular vision requires precise control over the stimuli presented to the left and right eyes. A popular technique is to segregate signals either temporally (frame interleaving), spectrally (using colored filters), or through light polarization. None of these segregation methods achieves perfect isolation, and so a degree of crosstalk is usually apparent, in which signals intended for one eye are faintly visible to the other eye. Previous studies have reported crosstalk values mostly for consumer-grade systems. Here we measure crosstalk for eight systems, many of which are intended for use in vision research. We provide benchmark crosstalk values, report a negative crosstalk effect in some LCD-based systems, and give guidelines for dealing with crosstalk in different experimental paradigms.

## Introduction

To study binocular vision, researchers rely on display systems that are capable of presenting separate images to the left and right eyes. In systems such as stereoscopes and virtual-reality headsets, the two images are optically segregated at all stages of presentation. Other systems separate the signals either spectrally, temporally, or using light polarization, requiring them to be demultiplexed at the eye, typically using active or passive goggles worn by the participant. Such systems suffer from the problem of crosstalk (or ghosting), where images intended for one eye are also faintly visible to the other eye (e.g., Pan, Lee, Huang, & Huang, 2010; Wang et al., 2011, 2012). In the following, we describe three common methods used for binocular stimulus presentation in a laboratory setting and how crosstalk can arise in these different systems.

### Temporal multiplexing with shutter goggles

Temporal multiplexing involves rapidly switching between left- and right-eye images on the display, and is typically combined with active shutter goggles that gate the appropriate image to each eye (though alternative methods have also been developed). The level of crosstalk depends on the efficiency with which the display type and the goggles can switch between the left- and right-eye images, and how closely they are synchronized. Display types include cathode-ray tubes (CRTs), liquid-crystal displays (LCDs), and Digital Light Processing (DLP) projectors. Crosstalk can arise here due to image persistence resulting from phosphor decay times in CRTs, pixel response rates in LCDs, or the image update method: top to bottom in CRTs and LCDs or at the same time in DLPs (Woods, 2012).

In addition, the properties of shutter goggles interact with these display types in different ways. Shutter goggles can be mechanical or use substances that can be rapidly switched from low to high transmittance, such as liquid crystal or ferroelectric materials. Here, crosstalk can arise from the optical performance of the material, the level of transmission in the opaque state, the efficiency of the synchronization to the display type, and the viewing angle through the goggles (Woods, 2012).

### Spectral multiplexing

A relatively inexpensive method for binocular stimulus presentation involves presenting the left- and right-eye images at different chromaticity, commonly red paired with either cyan, green, or blue. Participants view these images through anaglyph glasses with lenses of different passbands, such that different wavelengths pass through to either eye. An advantage to these systems is that they are MRI compatible, whereas active shutter goggles are not magnet safe. However, a disadvantage is that full-color images typically cannot be shown, and each eye receives an input that is not necessarily matched in its low-level properties (especially luminance and chromaticity).

Crosstalk in these systems results from a mismatch or an overlap in the spectral tuning of the stimulus and the filters. Achieving low levels of crosstalk using such systems can be challenging, in part due to the spectral bandwidth of sources such as CRT phosphors. Using narrow bandwidths for the source or the lenses can minimize crosstalk, as can tailoring the stimulus properties to suit the spectra of the lenses (Woods, Apfelbaum, & Peli, 2010).

### Polarized multiplexing

Polarization is an intrinsic property of light, where the electromagnetic wave can oscillate in more than one direction. A polarizing filter that switches between two polarizing states, paired with matching lenses, can be used to transmit images to each eye. Polarizers can be linear, where the images are encoded at 0° or 90°, or circular, where the polarization state is clockwise or anticlockwise. Polarized projection can be time sequential (similar to the temporal multiplexing already described) or simultaneous (Woods, 2012). Crosstalk in these systems depends on the quality of the polarizers, the extent to which the screen preserves the angle of polarization, and the orientation or decoding efficiency of the goggles. Head tilt is a cause of leakage, because a shift in the angle of the lenses results in suboptimal selectivity for the signals, permitting light intended for the other eye through the polarizer.

### Objectives

Despite their widespread use in vision research, objective methods for measuring crosstalk across these various systems have been reported relatively rarely (e.g., Woods, 2012; Woods et al., 2010), and typically for lower-performance consumer-focused devices (Weissman & Woods, 2011) rather than systems designed for use in research. Some studies report crosstalk for individual items of research-grade equipment, though it can be difficult to draw comparisons between systems when different methodologies are used across studies. In this article, crosstalk was assessed for eight stereoscopic display systems using a photometer, in order to provide benchmark measurements. We also report a measurement of contrast (rather than luminance) crosstalk (sometimes called gray-to-gray crosstalk; see, e.g., Chen, Ye, Huang, & Chen, 2015), as well as the existence of negative crosstalk on some LCD-based systems, in which the polarity of the ghost image is reversed. We provide recommendations for best practices for a range of different binocular experimental paradigms.

## Methods

### General procedure

We measured the luminance of a square target stimulus with a width 50% of the width of each display. Measurements were taken at 32 luminance levels on linearized (i.e., gamma-corrected) equipment. The background luminance and the luminance of the unstimulated channel (eye) were either (nominally) 0 or set to 50% of the maximum luminance. Readings were taken in a dark room with the photometer pointed through the right aperture (eye) of the goggles. The target was displayed to either the left or the right aperture. We also took a measurement of maximum luminance displayed binocularly, both with and without the goggles.

### Equipment

We used a Minolta LS-110 photometer (Minolta, Osaka, Japan) for all readings. These devices are widely used for performing gamma correction, and have a wide dynamic range. Measurements were performed manually for the following systems:

#### Clinton Monoray and ferroelectric goggles

The Clinton Monoray monitor is a customised MR2000HB-MED display (Richardson Electronics, LaFox, IL) using a special DP104 phosphor that has a very rapid decay time (∼0.4 ms, well below the frame duration of 8.33 ms at 120 Hz). This makes it ideal for stereo applications using frame interleaving, as the phosphor persistence should be minimal across successive frames. It was sold for many years by Cambridge Research Systems (CRS; Rochester, UK) before production ceased. However, it is still widely used in contemporary psychophysical studies of binocular luminance and contrast combination (Baker, Wallis, Georgeson, & Meese, 2012), binocular rivalry (Baker & Cass, 2013), dichoptic masking (Baker & Meese, 2007), and stereopsis (Georgeson, Yates, & Schofield, 2008). The Clinton is a monochrome monitor, the phosphor having a yellow hue.

Binocular separation was achieved using ferroelectric shutter goggles (FE-1; CRS) with a very rapid maximum switch time (much less than 1 ms). The switching of the goggles was synchronised with the refresh rate of the monitor to permit stable presentation of images to each eye. The system was controlled by a ViSaGe frame-store device (CRS) with a frame rate of 120 Hz. This meant that the effective refresh rate for each eye was 60 Hz due to the frame interleaving of the goggles, which is above the typical flicker-fusion frequency for photopic human vision (e.g., Simonson & Brozek, 1952). Code to display the target stimuli was written in MATLAB using the CRS VSG toolbox, running on a PC.

#### Clinton Monoray and liquid-crystal goggles

The second system was identical to the first, except that a pair of low-cost liquid-crystal shutter goggles (NeoTek, Pittsburgh, PA) was used instead of the ferroelectric variety. Liquid-crystal goggles have a much slower switch time, and tearing artifacts were apparent in the upper third of the display during testing (a consequence of the top-to-bottom raster scan). These goggles were included as a baseline for the more sophisticated systems, though in fact they performed somewhat better than expected across the lower region of the display.

#### Iiyama VisionMaster Pro and ferroelectric goggles

This system was the same as the first, except that the monitor was replaced with an Iiyama VisionMaster Pro 510 (Iiyama, Iiyama, Japan). This monitor lacks the fast phosphor of the Clinton Monoray, so we expected increased crosstalk due to luminance leakage from phosphor persistence. Again, this configuration was included for comparison with other systems.

#### VIEWPixx 3D with active shutter goggles

The VIEWPixx 3D (VPixx Technologies, Montreal, Canada) is an LCD panel with an LED backlight. It is a high-performance system intended to replace CRT monitors in vision-science applications. When paired with an active-shutter-goggle system (Nvidia 3D Vision, Nvidia, Santa Clara, CA), it is capable of stereo presentation at 120 Hz. This system was driven by a 2013 Mac Pro (Apple, Cupertino, CA), with code written in MATLAB using Psychtoolbox 3.0.11.

#### ViewSonic LCD panel with active shutter goggles

We tested a consumer-grade 3-D monitor (ViewSonic V3D245; ViewSonic, Brea, CA) intended for gaming and general computer use. It was paired with the Nvidia shutter goggles from the previous system, and the monitor had an inbuilt infrared transmitter to permit synchronization. Due to spatial nonuniformities and temporal nonlinearities, measurement of crosstalk in LCD systems is a complex issue that has been discussed more extensively elsewhere (e.g., Hong, 2012; Penczek, Boynton, & Kelley, 2013; Tourancheau et al., 2012), and our aim here was to provide comparisons to other systems using a common methodology rather than a detailed investigation.

#### Colored anaglyph filters

**Figure 1:**
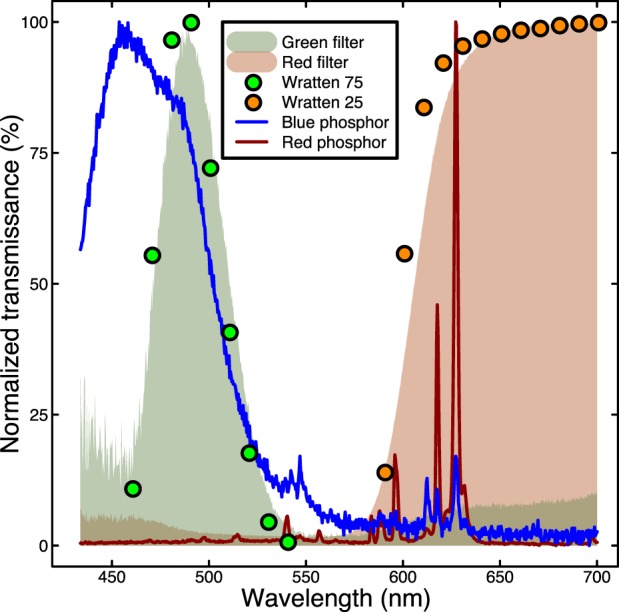
Properties of anaglyph filters and monitor phosphors, measured using a spectroradiometer. The shaded regions show the transmittances of red and green Wratten filters, which correspond well to published values (circles). The lines show the spectral properties of the blue and red phosphors of the Iiyama VisionMaster Pro monitor.

In numerous studies, binocular separation has been achieved using plastic colored filters (usually red for one eye and green or blue for the other) and tinting the stimuli in these colors. This anaglyph arrangement is notorious for causing substantial crosstalk (e.g., Woods, Yuen, & Karvinen, 2007), so it was included as a baseline condition. This system used the same Iiyama CRT display as an earlier system. The filters were narrowband Kodak gels (Wratten 75 and Stereo 25). We measured the monitor phosophor spectra and the filter transmittance spectra using a spectroradiometer (Jaz Spectrometer, Ocean Optics, Dunedin, FL). Figure 1 shows these measurements, along with published data on filter transmittance (Kodak, 1968) for comparison. Because the blue phosophor of the monitor overlapped less with the red filter than did the green phosophor, we made measurements through the red and green filters using the red and blue guns of the CRT. We averaged these qualitatively similar measurements to give an estimate of overall levels of crosstalk.

#### PROPixx projector with polarizing filter and passive goggles

The PROPixx (VPixx) is a high-performance DLP LED projector, capable of refresh rates of up to 500 Hz. It was paired with a DepthQ Polarization modulator (Lightspeed Design, Bellevue, WA), which polarized the light output by the projector on a frame-by-frame basis (quoted switch time of 0.05 ms), synchronized with the projector refresh rate (120 Hz). With projection onto a screen that maintains light polarization, binocular separation is obtained by using a pair of passive circularly polarized glasses, similar to those used in commercial cinemas. This combination of equipment can be used in MRI research, as no active hardware is required in the scanner room and the glasses contain no metal. This system was driven using the same computer hardware and software as the earlier VIEWPixx 3D system. Because the level of polarization depends on the viewing angle, we took measurements at 0°, 22.5°, and 45° relative to the normal of the plane of the screen, and tested several screen materials including Da-Lite 3D Virtual Grey and Virtual Black (Da-Lite, Warsaw, IN) for front and rear projection, respectively, and a rear-projection acrylic screen. We report values for the acrylic screen, though performance was similar for the other materials.

#### Optoma DLP projector with ferroelectric goggles

We also tested a commercially available DLP projector (Optoma EX785; Optoma Corporation, Taipei, Taiwan) in combination with the FE-1 shutter goggles. The projector was fitted with a wide-throw lens and driven at 120 Hz by a PC. It was projected onto a blank wall. Note that the wide-throw lens is responsible for the low pixel density of this display device relative to the others.

Information on the resolution, pixel density, and luminance of all eight display systems is given in Table 1.

**Table 1:**
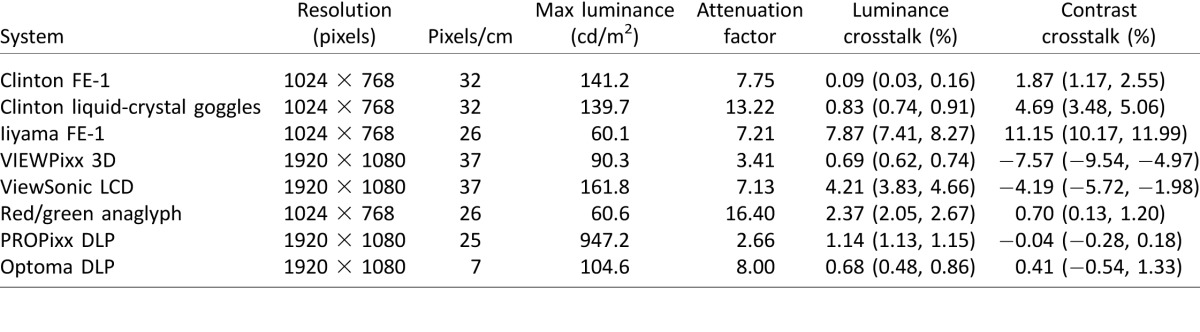
Details of display characteristics and summaries of crosstalk and attenuation. *Notes*: To determine the luminance recorded through the goggles, the displayed luminance can be divided by the attenuation factor. Errors on the crosstalk values are lower and upper 95% confidence intervals estimated by 1,000 bootstrap resamples of the data.

## Results

**Figure 2:**
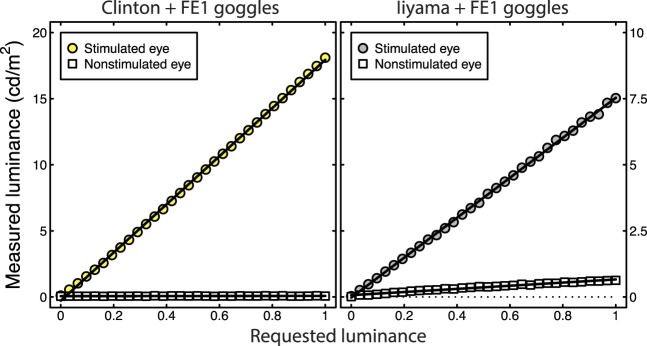
Illustrative luminance measurements from two systems, the Clinton (left) and Iiyama (right) CRT monitors. Circles represent measurements through the stimulated goggle, and squares show measurements through the nonstimulated goggle. In all cases, x-axis values are the requested luminance of the stimulated eye, and the nonstimulated eye had a requested luminance of 0. The solid lines are best-fit regression lines.

Figure 2 illustrates the patterns of measurements recorded for two extreme examples—the Clinton FE-1 system, which produced negligible crosstalk, and the Iiyama FE-1 system, which produced substantial crosstalk. As the only difference between these systems was the phosphor used in the monitor, the increased crosstalk is due to luminance bleed from phosphor persistence across frames. In both panels, the circles illustrate the condition in which squares of increasing luminance were measured through one shutter, with the other shutter viewing a dark screen. Measured luminance increased linearly, illustrating the success of our gamma-correction procedures. The white squares in both panels show the amount of luminance measured for the converse situation—where the photometer measured through a shutter showing only a dark screen, with targets of increasing luminance displayed through the other shutter. In an ideal stereo system, this condition would produce an entirely flat line, as is essentially the case for the Clinton FE-1 system shown in the left panel. However, substantial crosstalk was apparent with slower phosphors, with measured luminance increasing linearly at a rate of about 8% of that in the previous condition.

**Figure 3:**
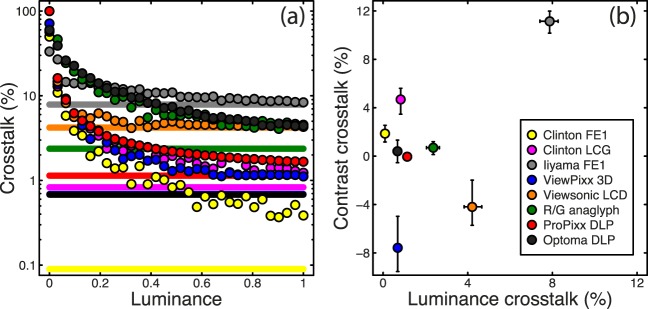
(a) Percentage crosstalk values for all eight systems as a function of requested luminance of the stimulated eye. Points show crosstalk values calculated by dividing the luminance measured through the unstimulated eye by the luminance measured through the stimulated eye, expressed as a percentage. Lines show the crosstalk estimate derived from the ratio of fitted regression slopes for the two eyes. Note the logarithmic scaling on the y-axis. (b) Contrast crosstalk plotted against luminance crosstalk; negative contrast crosstalk is apparent for some systems. Error bars represent 95% confidence intervals calculated by repeating the regression procedure 1,000 times on resampled (bootstrapped) data.

To quantify this luminance crosstalk, we took the ratio of luminances in these two conditions and expressed it as a percentage. The points in Figure 3a show this measure for all eight systems. There was substantial variation across equipment, with some systems (e.g., the Clinton FE-1 system and the VIEWPixx) producing negligible crosstalk and others (e.g., anaglyph glasses and the Iiyama monitor) producing very substantial crosstalk.

Ratios were inflated at low luminances owing to the small values involved and possible light scatter from other parts of the display (a nonzero black level). Therefore, to produce a single general estimate of luminance crosstalk for a given system, we fitted regression lines to the luminance functions (see Figure 2 for examples) and took the ratio of fitted slope (regression beta) parameters as an alternative measure of crosstalk. We present these values expressed as percentages in Table 1 and in the horizontal lines of Figure 3a. This method corresponded reasonably well to average crosstalk estimates across the higher luminance regime (i.e., points above a requested luminance of 0.5 in Figure 3a).

Several individual systems are worthy of mention. First, it is clear that the fast-decaying phosphor of the Clinton CRT conferred a substantial benefit over typical CRT phosphors such as those used in the Iiyama monitor, reducing the crosstalk by more than a factor of 10 and delivering the lowest crosstalk values we measured. Both the VIEWPixx and PROPixx devices produced minimal crosstalk (around 1%), which was less than anaglyph filters (>2%) or a consumer-grade LCD monitor (ViewSonic, >4%). We also tested some more broadband anaglyph filters, of the type often provided free in cinemas and on breakfast-cereal boxes in the 1980s. These produced enormous amounts of crosstalk (>40%) and would not be suitable for use in any serious laboratory setting. We also note that the narrowband filters we used here substantially attenuated the monitor luminance (by a factor of >16), rendering images much dimmer than might be desirable in many experiments.

We also noted that the amount of crosstalk for the PROPixx projector increased substantially with viewing angle. This is because the circular polarization used in the passive glasses is dependent on the relative angle between the display screen and the lens of the glasses. Crosstalk increased from around 1% at 0° relative angle to 7% at 23° and around 20% at 45°. Additionally, rotating the goggles produced chromatic abberations in the image, presumably as a consequence of the color wheel in the DLP projector or the wavelength dependency of the polarizers (see Woods et al., 2010). Since this technology is intended for use in MRI scanners, it is important to achieve good alignment between the plane of the goggles and the plane of the screen.

### Contrast crosstalk

**Figure 4:**
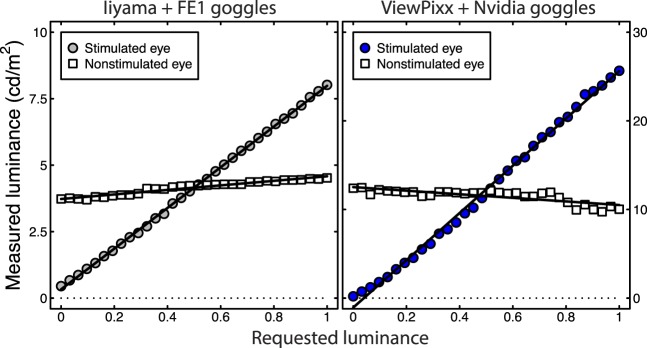
Illustrative contrast crosstalk measurements from two systems. Circles represent measurements through the stimulated goggle, and squares show measurements through the nonstimulated goggle. The Iiyama CRT monitor (left) shows positive crosstalk (the slope of the nonstimulated eye is positive). The VIEWPixx LCD monitor (right) shows negative crosstalk (the slope of the nonstimulated eye is negative). In all cases, x-axis values are the requested luminance of the stimulated eye, and the nonstimulated eye had a requested luminance of 0.5. The solid lines are best-fit regression lines.

In many experimental situations, stimuli are presented against a mean-luminance background. Under these circumstances, it is helpful to estimate the amount of crosstalk for luminance contrast relative to this background level. Measuring luminance for an eye (or shutter) viewing mean luminance while the luminance to the other eye is varied (see Figure 4 for examples) permits such an estimate (sometimes called the gray-to-gray crosstalk; see Chen et al., 2015).

The percentage of contrast crosstalk was again defined by taking the ratio of slopes of nonstimulated and stimulated eyes and expressing it as a percentage. This is approximately equivalent to the percentage Michelson contrast that would be expected to bleed through to the nonstimulated eye if the stimulated eye were shown a 100% contrast grating. Inspired by Xia, Li, Cui, Chen, and Teunissen (2013), we also calculated contrast crosstalk as 100*(*L*_max_ − *L*_min_)/(*L*_max_ + *L*_min_), where the maximum and minimum luminance readings were the extreme left- and rightmost white squares in each panel of Figure 4, and obtained comparable values. Estimates of contrast crosstalk are given in Table 1. As with the luminance crosstalk measure, an ideal system would have zero contrast crosstalk, as the maximum and minimum readings would be the same and the line would be flat.

Figure 3b shows contrast crosstalk plotted against luminance crosstalk for the eight systems tested. The Pearson's correlation coefficient between the two measures was 0.57, though this was not significant. However, the worst system we tested (the Iiyama CRT monitor) had both the highest luminance and contrast crosstalks. Of particular note is that two systems produced strongly negative contrast crosstalk (see also Barkowsky, Tourancheau, Brunnström, Wang, & Andrén, 2011; Kang, Lee, Lee, & Song, 2012). These were the VIEWPixx and ViewSonic LCD monitors, shown by the blue and orange points in Figure 3b. Example luminance measurements for the VIEWPixx are given in Figure 4b, and it is apparent that the function for the nonstimulated eye has a substantially negative slope. Possible explanations for this result will be discussed in the following section.

## Discussion

We measured crosstalk for eight stereoscopic systems. There was wide variation in luminance crosstalk, from less than 0.1% to over 7%. We also report measures of contrast crosstalk (sometimes called gray-to-gray crosstalk; see Chen et al., 2015; Woods, 2012), which for some LCD-based systems could be negative. We now discuss possible explanations for this finding, and then consider the implications for various levels of crosstalk for different psychophysical binocular paradigms.

### Negative crosstalk

The phenomenon of negative crosstalk was substantial and could easily be observed by displaying a high contrast image to one shutter and perceiving a low-contrast, polarity-inverted version of the same image through the other shutter. It occurred only for the two displays that use LCD technology (the VIEWPixx and ViewSonic monitors). A peculiarity of LCD technology is that the voltage of each pixel is typically inverted on alternate frames to prevent a DC voltage buildup that could damage the liquid crystal and cause it to perform in a suboptimal fashion (e.g., Yoo, 2003). However, during frame-interleaved stereo, if the luminances shown to the two eyes are very different, this DC-balancing strategy will fail (because all of the positive-voltage frames will have one luminance and all of the negative-voltage frames will have a very different luminance). This may be responsible for changing the displayed luminance of one eye in the opposite direction to that shown in the other eye (rather like a temporal version of the adjacent-pixel nonlinearity on CRT displays).

We contacted the manufacturers of the VIEWPixx display, who confirmed this assessment of the phenomenon of negative crosstalk. The system has a feature that is designed to resolve this problem (using the command *EnableVideoLcd3D60Hz*); however, we had already activated this function before making our measurements, and we still observed negative crosstalk effects. Previous studies have suggested that negative crosstalk could be due to overzealous crosstalk-canceling algorithms (Barkowsky et al., 2011), which also appears plausible. Indeed, LCD technology involves a number of complex processes to present images, any combination of which might contribute to crosstalk, and this will likely differ across different displays. Since crosstalk is an inevitable feature of all goggle-based stereo systems, we now discuss some ways to mitigate its effects for different types of experiment.

### Equipment recommendations for different paradigms

#### Binocular rivalry and continuous flash suppression

A very active area of research into binocular vision involves presenting conflicting stimuli to the two eyes to induce binocular rivalry (e.g., Blake, Brascamp, & Heeger, 2014) or showing a high-contrast dynamic stimulus to one eye in order to suppress images shown to the other eye (continuous flash suppression; Tsuchiya & Koch, 2005). For the high-contrast stimuli typically used in these studies, crosstalk of under around 2% is unlikely to have a substantive effect on experimental results, because monocular masking (e.g., Foley, 1994) from the high-contrast image in the other eye will render the crosstalk image invisible. However, precise quantitative studies in which contrast is manipulated as a parameter may wish to avoid systems that exhibit higher levels of crosstalk (especially anaglyph filters, which are still used surprisingly often in rivalry research). In particular, it is conceivable that positive crosstalk will encourage binocular fusion of the real and crosstalk images, which might affect the alternation rate of rivalry and perhaps encourage mixed percepts (consider the case where crosstalk was 100%: Each eye would see both images at the same contrast, and rivalry would not occur). For experiments where this is of critical importance, a mirror stereoscope or virtual-reality goggles are safer options.

#### Detection, masking, and matching

Measuring monocular or binocular detection thresholds should not be greatly affected by crosstalk, since the contrasts used are very small and any crosstalk images will be far below threshold. However, masking experiments in which a high-contrast pedestal mask is shown to one eye and a target detected in the other might well be affected. Woods et al. (2010) have demonstrated that when the masked eye is patched, crosstalk from a high-contrast mask can influence detection in the other eye, producing facilitation from a pedestal effect. Negative crosstalk would be expected to cancel with the signal and cause masking. In a typical experimental situation, the masked eye is not occluded, and detection thresholds are greatly elevated by such a high-contrast mask for physiological (rather than optical) reasons (i.e., Legge, 1979). However, in the case of severe unilateral insensitivity (for example in amblyopia, where the amblyopic eye might be insensitive to the mask), it is conceivable that masking effects from a crosstalk image might be misconstrued as a physiological influence from the masked eye.

Contrast- or luminance-matching paradigms might be more severely affected by crosstalk. Consider an experiment in which the contrast (or luminance) of a monocular target is compared with that of a binocular target (e.g., Anstis & Ho, 1998; Baker et al., 2012). In a situation of high positive contrast crosstalk, a monocular target might appear reduced in contrast relative to a binocular target, as it will lose some of its contrast to the other eye (which will make a negligible contribution to perception). In a situation of high negative contrast crosstalk, the reverse should occur, as the binocular stimulus will partially cancel itself out through the negative-crosstalk images. If precise quantitative comparisons across monocular and binocular responses are required from such a paradigm, it would be advisable to avoid any systems subject to crosstalk.

#### Rich stereoscopic stimuli

Contemporary stereoscopic displays are designed to display 3-D media such as movies and computer games. It therefore seems unlikely that the sometimes-strong crosstalk seen with LCD panels (see Table 1 and Figure 4) should have a substantive effect on rich stereoscopic media. Nevertheless, individuals often experience visual discomfort from prolonged viewing (e.g., Lambooij, Ijsselsteijn, Fortuin, & Heynderickx, 2009). One possibility is that crosstalk images could interfere with stereo correspondence, making it more difficult to achieve binocular fusion and perhaps contributing to discomfort or fatigue. This could be explicitly tested by simulating crosstalk artificially in a relatively crosstalk-free system (i.e., by adding a weak version of one eye's image to the other eye and vice versa). However, we suspect (at least anecdotally based) that the mismatch between accommodation and vergence is more severe for stereoscope and virtual-reality systems than it is for arrangements such as those discussed here which permit natural viewing of a distant display. These systems might therefore be preferable for minimizing visual discomfort during viewing of realistic stereo stimuli for long periods of time.

#### Sparse stereoscopic stimuli

Creating the illusion of depth using sparse stereoscopic stimuli composed of lines or dots has a long and illustrious history (see Howard & Rogers, 2002). For bright stimuli shown against a dark background, positive crosstalk could produce visible ghost images that interfere with depth perception (Tsirlin, Wilcox, & Allison, 2012; Watanabe, Ujike, Penczek, & Boynton, 2014). However, these should be easily apparent and could therefore be detected during stimulus development and perhaps avoided by using a higher-mean-luminance background that will mask the crosstalk images. Conversely, systems subject to negative crosstalk should be relatively immune to crosstalk effects on a dark background (as luminance cannot drop below zero) but might suffer them with a high mean luminance. The crosstalk properties of a given display should therefore factor into the experimenter's choice of stimulus parameters, in such a way that the effects on perception are minimized.

### Canceling crosstalk

If the crosstalk of a given system has been characterized, its effects might be mitigated by adding an image to the affected eye that is the negative of the expected crosstalk (so-called ghostbusting; see Konrad, Lacotte, & Dubois, 2000). For color images, this must be done separately for each color channel (and on a CRT the three color channels may have different amounts of crosstalk depending on the decay properties of their individual phosphors). We note that this technique is not feasible for positive crosstalk of bright stimuli against a black background, as the display would lack the dynamic range to compensate for the crosstalk. Under some circumstances this method can alleviate problems with crosstalk, though it would ideally require greater dynamic range than is typically available on 8-bit graphics cards to be optimally realized.

## Conclusions

Luminance and contrast crosstalk were measured for a range of stereoscopic systems based on different display technologies. All systems exhibited some level of crosstalk, and we report the presence of negative contrast crosstalk for some displays that use liquid-crystal technology. Crosstalk will affect various binocular experimental paradigms to differing extents, and our hope is that the values reported here will be used to guide the choice of display systems for future studies and as benchmark values against which new technologies (i.e., organic LED displays) might be compared. We describe a straightforward methodology for measuring crosstalk that can be easily implemented in order to assess the levels of luminance and contrast crosstalk for a given system.

## Supplementary Material


