# Supplementary figures and images for: Measurement of crosstalk in stereoscopic display systems used for vision research
Source: J Vis. 2016 Dec 15;16(15):14. doi: 10.1167/16.15.14 (PMC5172160; doi:10.1167/16.15.14)

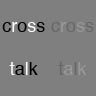

Supplement: Supplementary file 1 [file JOV-05292-2016-s01-ICON.jpg]
